# ESA VIVALDI Dry Immersion Microgravity Simulations Induce Increases in Immune Biomarkers Associated With Physical and Psychological Stress, and Sex‐Specific Factors

**DOI:** 10.1096/fj.202502198R

**Published:** 2025-09-02

**Authors:** Pauline Jacob, Adrien Robin, Nastassia Navasiolava, Marc‐Antoine Custaud, Stéphanie Ghislin, Marie‐Pierre Bareille, Rebecca Billette De Villemeur, Inês Antunes, Angelique Van Ombergen, Guillemette Gauquelin‐Koch, Jean‐Pol Frippiat

**Affiliations:** ^1^ UR SIMPA, Stress Immunity Pathogens Laboratory, Faculty of Medicine University of Lorraine Vandœuvre‐lès‐Nancy France; ^2^ CRC, CHU Angers, Inserm, CNRS, MITOVASC, Equipe CARME, SFR ICAT University of Angers Angers France; ^3^ Department of Aerospace Engineering Texas A&M University College Station Texas USA; ^4^ Institute of Space Physiology and Medicine MEDES Toulouse France; ^5^ Telespazio Belgium S.R.L. for the European Space Agency Noordwijk the Netherlands; ^6^ University of Antwerp Antwerp Belgium; ^7^ European Space Agency Noordwijk the Netherlands; ^8^ Centre National d'Etudes Spatiales Paris France

**Keywords:** astronaut health, biomarker, deep‐space missions, ground‐based model, monitoring, peripheral blood cells, physical stress, vascular system

## Abstract

With future manned space projects involving missions of unprecedented duration, multisystem deconditioning induced by spaceflight could seriously affect the well‐being and health of astronauts. Safe and easily determined in‐flight biomarkers are therefore needed to monitor health status. In this study, we simulated space deconditioning with a 5‐day dry immersion (DI) of 18 healthy women and 19 healthy men and evaluated the effects of this protocol on three biomarkers: the neutrophil‐to‐lymphocyte ratio (NLR), the granulocyte‐to‐lymphocyte ratio (GLR) and the platelet‐to‐lymphocyte ratio (PLR). Increases in all three ratios were observed in both men and women, as also observed at the end of a space mission or after exposure to simulated microgravity. These increases were associated with physical and psychological stress in both sexes. Furthermore, our work suggested a positive link between NLR increase and cardiovascular system alteration in women, whereas in men, there would be a positive relationship between NLR, GLR, PLR, and inflammation. Thus, in addition to physical and psychological stress, sex‐specific factors could contribute to increases in NLR, GLR, and PLR ratios during DI. As for the increase in PLR, it did not predict the development of long‐lasting immune diseases during DI, in contrast to 2 months of head‐down tilt bed rest (HDBR), another spaceflight analog. These data show that the NLR, GLR, and PLR ratios are promising biomarkers that deserve further study to determine the relationships between their increase and microgravity‐induced deconditioning. These dry immersion investigations are registered at clinicaltrials.gov as NCT05043974 for women and NCT05493176 for men.

## Graphical abstract

Exposure of healthy men and women to 5 days of dry immersion (DI), a terrestrial analogue of microgravity, induced elevated neutrophil‐to‐lymphocyte (NLR), granulocyte‐to‐lymphocyte (GLR), and platelet‐to‐lymphocyte (PLR) ratios that correlated with physical and psychological stress in both sexes. Furthermore, positive links between increased NLR and cardiovascular alteration in women and increased NLR, GLR, PLR, and inflammation in men were noted, suggesting that sex‐specific factors may also contribute to these increases. DI model drawing credit: Robin et al. [8].

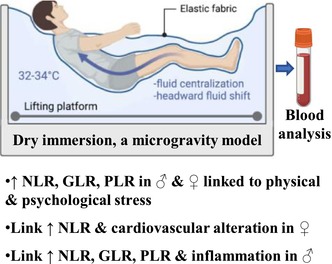

**Abbreviations**
AIP: Atherogenic index of plasma
BDC: Baseline data collection
DI: Dry immersion
ESA: European Space Agency
GLR: Granulocyte‐to‐lymphocyte ratio
HARV‐RWV: high‐aspect rotating wall vessel
HDBR: Head‐down tilt bed rest
hsCRP: High‐sensitivity C‐reactive protein
ISS: International Space Station
NLR: Neutrophil‐to‐lymphocyte ratio
PLR: Platelet‐to‐lymphocyte ratio
RPM: Random positioning machine
SLS‐2: Space Life Sciences (SLS)‐2 mission
TCRβ: Beta chain of the T‐cell receptor
UFC: Urinary free cortisol
VEGF: Vascular endothelial growth factor
VEGFR‐1: Vascular endothelial growth factor receptor‐1

## 1 Background

Manned spaceflight projects are increasing, as evidenced by the Gateway program, an international collaboration aimed at establishing humanity's first space station around the Moon and paving the way for the first human missions to Mars along with the development of space tourism, which is attracting a growing number of companies. However, during spaceflight, the body is subjected to a combination of socioenvironmental and physical stressors that induce numerous physiological deregulations, such as muscle atrophy, bone demineralization, cardiovascular and metabolic dysfunctions, altered cognitive processes, and reduced immunological competence [1].

Implementing such ambitious programs therefore requires the preservation of astronauts' well‐being and health. To this end, it is mandatory to identify safe, cost‐effective, and easily determined in‐flight biomarkers to monitor health status, determine when countermeasures should be applied to promote recovery, prevent the development of disease, and track responses to treatment during space missions.

Previous studies have suggested the neutrophil‐to‐lymphocyte ratio (NLR), the granulocyte‐to‐lymphocyte ratio (GLR), and the platelet‐to‐lymphocyte ratio (PLR) as biomarkers for future in‐flight immune health monitoring [2, 3]. Changes in these ratios have also been associated with aging [4, 5, 6], which is interesting given that spaceflight may accelerate aging processes [7].

In this explorative study, we determined these three ratios in 19 men and 18 women subjected to 5 days of dry immersion (DI) during the European Space Agency (ESA) VIVALDI 1 and 2 studies. DI involves whole‐body thermoneutral immersion with a waterproof film separating the subject from the water, creating a supportless environment characterized by buoyancy‐induced gravitational unloading, constant hydrostatic compression of superficial tissues with swift fluid centralization and headward fluid shift, constant ambient temperature, constant body position, and constant physical inactivity with an acute decrease in postural muscle load and body movements [8]. These factors are responsible for the multisystem deconditioning that occurs during this microgravity simulation.

The obtained results were compared with those observed during head‐down tilt bed rest (HDBR), another spaceflight analog platform simulating microgravity but with differing conditions and spaceflight. We also investigated whether these ratios are different in men and women because there are sex‐related differences in hormones, stress, and immune responses as well as in cardiovascular, immunologic, sensorimotor, musculoskeletal, reproductive, and behavioral adaptations to spaceflight [9, 10, 11]. Finally, we searched for correlations with stress markers, soluble markers of endothelial status, as well as the atherogenic index of plasma (AIP), because AIP and elevated NLR and PLR are predictive of cardiovascular disease risk [12, 13, 14] and microgravity has been shown to induce an aging‐like cardiovascular deconditioning [15]. Correlations with high‐sensitivity C‐reactive protein (hsCRP) were also explored to investigate the contribution of inflammation.

## 2 Materials and Methods

### 2.1 Ethics

The VIVALDI 1 and 2 exploratory studies were carried out in accordance with the World Medical Association Helsinki Declaration of 1975 (revised in 2008) and were approved by the National Ethics Committee (CPP Ile de France II, ID‐RCB 2021‐A00705‐36 for VIVALDI 1 performed on women, CPP Ile de France VII, ID‐RCB 2022‐A00881‐42 for VIVALDI 2 performed on men). These trials are registered at clinicaltrials.gov as NCT05043974 for VIVALDI 1 and NCT05493176 for VIVALDI 2.

Each trial involved the recruitment of 20 young healthy participants. All were informed about the experimental procedures and provided written consent. One female participant left the protocol on the first day of immersion due to a technical issue (a technical problem with the bath lifting platform, which could not be fixed quickly without emptying the bath, leading to the decision to stop the study for this participant), and another could not be included for regulatory reasons (this participant unexpectedly was still in the exclusion period following participation in a previous unrelated clinical trial). One male participant withdrew from the study on the third day of immersion due to severe back pain. These three subjects were excluded from the analysis.

### 2.2 Dry Immersion

Eighteen women (mean ± SEM at baseline, age: 29 ± 1; height: 164.8 ± 1.4 cm; weight: 59 ± 1.5 kg) and 19 men (age: 28 ± 1; height: 176.6 ± 1 cm; weight: 72 ± 1.5 kg) were ultimately included in the VIVALDI 1 and 2 studies, respectively. These subjects were confirmed to be healthy by a comprehensive clinical assessment, including a detailed medical history and complete physical examination. All were nonsmokers and were not taking any drugs or medications. The VIVALDI 1 and 2 studies were conducted at the Space Clinic of the Institute of Space Medicine and Physiology (MEDES‐IMPS) in Toulouse, France, between September 2021 and November 2022. These studies were sponsored by the European Space Agency in collaboration with the French National Space Agency (CNES).

The participants arrived in the evening of the fifth day before exposure to DI and left in the morning of the third day after DI exposure. The experimental protocol included 4 days of ambulatory baseline measurements before immersion (baseline data collection, BDC‐4 to BDC‐1), 5 days of DI (DI1 to DI5), and 2 full days of ambulatory recovery (R0, R+1). This 5‐day DI was designed to study the effects of the early phase of adaptation to microgravity. For a relatively short duration, this model can faithfully reproduce most of the physiological effects of actual microgravity [8, 16].

VIVALDI 1 and 2 participants were subjected to a strict DI protocol (they were not allowed to sit or stand up at all) to be close to what happens during a spaceflight. However, daily hygiene, weighing, and some specific measurements (as several scientific protocols were carried out in parallel during VIVALDI 1 and 2) necessitated extraction from the bath with an integrated lifting platform. During these brief out‐of‐bath periods, the participants maintained the 6° head‐down position. The water temperature was continuously maintained in a thermoneutral environment (32°C–34°C). The light‐off period was set at 23:00 to 07:00. On each experimental day, meals were identical for all participants.

### 2.3 Blood Sampling

Venous blood samples were collected in the morning before breakfast 4 days and 1 day before DI exposure (BDC‐4 and BDC‐1), in the evening of the first day of DI (DI1), in the morning of the third and fifth days of DI (DI3 and DI5), and in the morning of recovery day 1, approximately 22 h after completing DI (R+1).

### 2.4 Blood Analyses

Blood cell counts were determined using an Advia 2120 automated hematology analyzer (Siemens Healthcare Diagnostics, Deerfield, IL, USA). The NLR, GLR, and PLR ratios were calculated from the neutrophil, granulocyte, platelet, and lymphocyte counts provided by these blood count measurements. Plasma vascular endothelial growth factor (VEGF), VEGF‐receptor 1 (VEGF‐R1), and E‐selectin were quantified by ELISA using human VEGF, VEGFR1/Flt‐1, and sE‐Selectin/CD62E Quantikine ELISA kits (R&D Biosystems, Minneapolis, MN, USA). The concentrations of high‐sensitivity C‐reactive protein (hsCRP), triglycerides (TGs) and high‐density lipoprotein (HDL) cholesterol were determined via immunoturbidimetry, enzymatic techniques, and catalase elimination, respectively (Advia Chemistry XPT systems, Siemens). From TG and HDL cholesterol molar concentrations, the atherogenic index of plasma (AIP), a marker of lipoprotein particle size, was calculated as the log (TG/HDL).

### 2.5 Questionnaires

General discomfort and back pain (morning and evening) and the quality of night sleeping (morning) were assessed daily from BDC‐4 to R+1 using a 0 to 10 visual analog scale [8]. Psychological state was estimated using the General Health Questionnaire GHQ‐28 in the evening at baseline, DI4, R0, and R+1. Subjects assessed their current state by comparing it with their usual state. GHQ consists of 28 items; 7 phrased in a positive way (e.g., Do you feel perfectly well and in good health?) and 21 phrased in a negative way (e.g., Do you feel constantly under strain?). For positive items, the following scale was used: 1 = more than usual, 2 = as usual, 3 = less than usual, 4 = much less than usual. For 18 negative items, the following scale was used: 1 = not at all, 2 = not more than usual, 3 = a little more than usual, 4 = much more than usual. For the remaining 3 items, other scale types were used. GHQ score was calculated in a traditional manner, by assigning a score of 0 for choices 1 and 2 and a score of 1 for choices 3 and 4, for all 28 items (0‐to‐28 scale).

### 2.6 Quantification of Urinary Free Cortisol

Twenty‐four‐hour urine collection started in the morning with the 2nd void and ended after the first void the following day. All samples were pooled into a storage container kept at +4°C. For each 24 h pool, aliquots were prepared and frozen at −80°C. Urinary free cortisol (UFC) was quantified via liquid chromatography–tandem mass spectrometry (Applied Biosystems/MDS Sciex Api 3000 triple quad mass spectrometer).

### 2.7 Statistics

The effects of DI and test days on NLR, GLR, PLR, mean of general discomfort, mean of back pain, sleep quality, UFC, and hsCRP of men and women were determined via repeated‐measures one‐way ANOVA with the Geisser–Greenhouse correction in combination with a *post hoc* Tukey's multiple comparison test or, when some data were missing, with a Geisser–Greenhouse‐corrected linear mixed effects model using the restricted maximum likelihood method in combination with *post hoc* Tukey's multiple comparison test. The effects of sex on NLR, GLR, and PLR data were determined via repeated‐measures two‐way ANOVA with the Geisser–Greenhouse correction in combination with *post hoc* Sidak's multiple comparison test or, when some data were missing, with a Geisser–Greenhouse‐corrected linear mixed effects model using the restricted maximum likelihood method in combination with *post hoc* Sidak's multiple comparison test. GHQ‐28 data were analyzed via repeated‐measures one‐way ANOVA with the Geisser–Greenhouse correction in combination with a Bonferroni *post hoc* to determine differences with the baseline data collection point or, when some data were missing, with a Geisser–Greenhouse‐corrected linear mixed effects model using the restricted maximum likelihood method in combination with a Bonferroni *post hoc*. Relationships between variables of interest were examined using the Pearson correlation coefficient for normally distributed data or the Spearman correlation coefficient for nonnormally distributed data (one‐tailed *p* value). The data were analyzed via GraphPad Prism 10.2.3 (Boston, MA, USA). An adjusted *p* value < 0.05 was considered statistically significant. The data are shown as the mean ± SEM.

## 3 Results

The DI protocol used in this study included 4 days of ambulatory baseline measurements before immersion (baseline data collection, BDC‐4 to BDC‐1), 5 days of DI (DI1 to DI5), and 2 full days of ambulatory recovery (R0, R+1).

### 3.1 Dry Immersion Induces Increases in NLR, GLR and PLR

**FIGURE 1:**
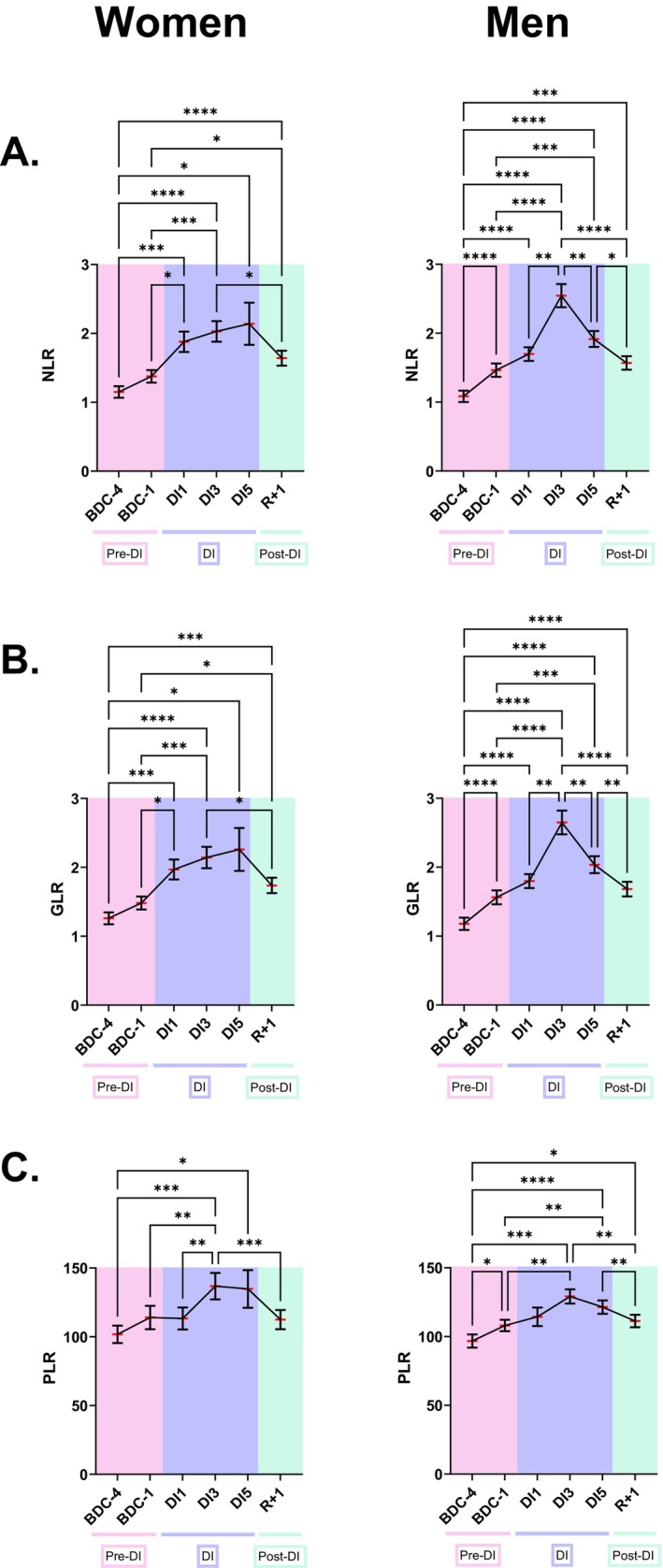
Evolution of the NLR (A), GLR (B) and PLR (C) ratios before, during and after five days of dry immersion in women (*n* = 18) and men (*n* = 19). The data are shown as mean ± SEM. Statistically significant differences were revealed via repeated‐measures one‐way ANOVA or linear mixed effects model analysis when some data were missing, both of which were followed by a Tukey *post hoc* test. *p < 0.05; **p < 0.01; ***p < 0.001; ****p < 0.0001. BDC, baseline data collection (pink); DI, dry immersion (blue); R, recovery (green).

A progressive increase in the NLR, GLR, and PLR ratios was observed from BDC‐1 in women: +47% ± 63% (mean ± SEM) (*p* = 0.0003) for NLR, +45% ± 65% (*p* = 0.0003) for GLR, and +20% ± 13% (*p* = 0.0017) for PLR at DI3 compared with BDC‐1 (Figure 1). No statistically significant differences in the NLR, GLR, or PLR values between BDC‐4 and BDC‐1 were noted in women. After DI3 to DI5, female NLR, GLR, and PLR ratios decreased, but did not reach, at R+1, the basal level observed at BDC‐4 and BDC‐1, except for PLR. We also noted a progressive increase in the NLR, GLR, and PLR ratios for men, but from BDC‐4 onward. These three ratios were statistically greater at BDC‐1 in men than at BDC‐4: +35% ± 17% (mean ± SEM) (*p* < 0.0001) for NLR, +33% ± 14% (p < 0.0001) for GLR, and + 12% ± 14% (*p* = 0.0173) for PLR at BDC‐1. After DI3, the NLR, GLR, and PLR ratios decreased in men, but did not return to the basal level observed at BDC‐4. The very similar evolution of NLR and GLR in women and men occurred because basophil and eosinophil levels are lower in peripheral blood than are neutrophil levels, which represent 50%–70% of the total leukocyte population. The NLR and GLR ratios were investigated so that at least one of these ratios could be determined using the hardware that will be used to perform on‐orbit biomedical analyses during future long‐duration space missions.

We also noted that the NLR and GLR ratios appeared to increase slightly more during DI in men than in women. At DI‐3, the mean male NLR and GLR values were approximately 25% greater than those for women (+25% ± 13% and +25% ± 11%, respectively), but these differences were not statistically significant at any time point, as determined via repeated‐measures two‐way ANOVA or mixed effects model analysis followed by a Sidak *post hoc* test.

**FIGURE 2:**
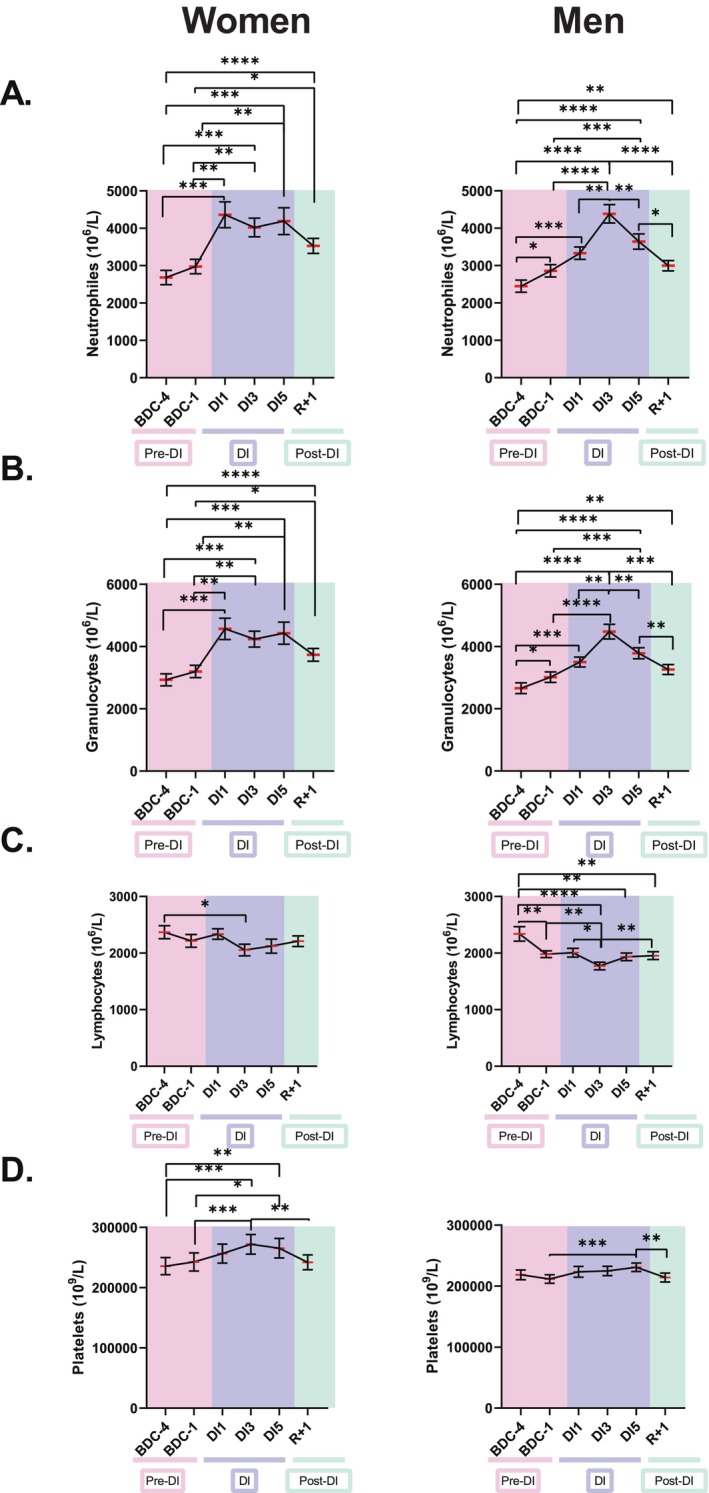
Evolution of neutrophil (A), granulocyte (B), lymphocyte (C) and platelet (D) counts before, during and after five days of dry immersion in women (*n* = 18) and men (*n* = 19). The data are shown as mean ± SEM. Statistically significant differences were revealed via repeated‐measures one‐way ANOVA or linear mixed effects model analysis when some data were missing, both of which were followed by a Tukey's *post hoc* test. *p < 0.05; **p < 0.01; ***p < 0.001; ****p < 0.0001. BDC, baseline data collection (pink); DI, dry immersion (blue); R, recovery (green).

The increases in NLR and GLR were due to an increase in neutrophils and granulocytes at DI1, DI3, and DI5 in women, a peak in these cells at DI3 in men, a slight decrease in lymphocytes at DI3 in women, and a greater decrease in lymphocytes at DI3 in men (Figure 2). The increases in PLR were due to an increase in platelets at DI3 and DI5 in women, and a decrease in lymphocytes at DI3 and DI5 in men.

Taken together, these data reveal progressive increases, followed by decreases, in NLR, GLR, and PLR ratios during DI due to changes in the concentration of both innate immune cells (neutrophils and granulocytes increased) and adaptive immune cells (lymphocytes decreased).

### 3.2 Relationships Between NLR, GLR, PLR and DI‐Associated Stress

**FIGURE 3:**
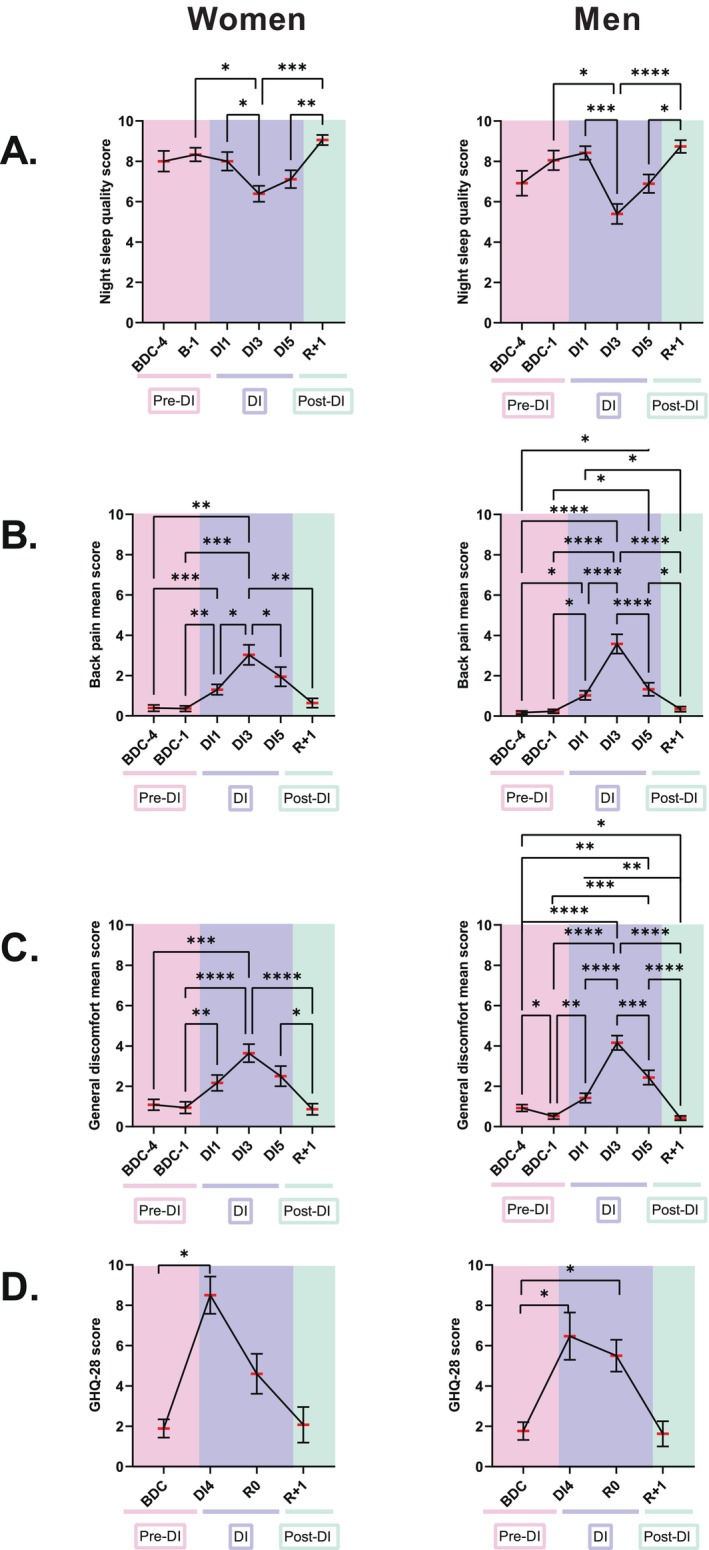
Evolution of the night sleep quality score (A), back pain mean score (B), general discomfort mean score (C) and GHQ‐28 score (D), used to estimate psychological state, in women (*n* = 18) and men (*n* = 19) exposed to 5 days of dry immersion. The morning and evening back pain and discomfort scores were averaged to obtain a daily score. The data are shown as mean ± SEM. Statistically significant differences were revealed via repeated‐measures one‐way ANOVA or linear mixed effects model analysis when some data were missing, followed by a Tukey or Boneferroni *post hoc* test. *p < 0.05; **p < 0.01; ***p < 0.001; ****p < 0.0001. BDC, baseline data collection (pink); DI, dry immersion (blue); R, recovery (green).

Jacob et al. [3] previously suggested that increases in the NLR and GLR ratios could be biomarkers of physical stress. Thus, we assessed DI‐induced biomechanical stress by studying the effects of this protocol on night sleep quality, back pain, and general discomfort. A statistically significant decrease in the night sleep quality score, which coincided with increases in back pain and general discomfort during DI, was observed in both women and men (Figure 3). Then, we searched for correlations between the evolution of the NLR, GLR, and PLR ratios and those of these three markers of DI‐induced biomechanical stress. Out of the 18 possible relationships (9 for women and 9 for men), 15 were statistically significant (Table 1). Eleven positive correlations (5 moderate and 6 weak) with back pain and general discomfort mean scores and four negative correlations (2 moderate and 2 weak) with night sleep quality scores were observed. These observations support the existence of a link between physical stress and changes in NLR, GLR, and PLR.

**TABLE 1:** Relationships between the NLR, GLR, and PLR ratios and DI‐induced biomechanical stress (night sleep quality score, back pain mean score, general discomfort mean score), psychological state (GHQ‐28 score), stress (UFC), inflammation (hsCRP), the atherogenic index of plasma (AIP) and endothelial markers (E‐selectin, VEGFR‐1, and VEGF).

	Night sleep quality score	Back pain mean score	General discomfort mean score	GHQ‐28 score	UFC (μg/24 h)	hsCRP (mg/L)	AIP	E‐selectin (ng/mL)	VEGFR‐1 (pg/mL)	VEGF (pg/mL)
NLR	*r* = −0.17 * ***r* = −0.30** ***	*r* = 0.24 ** ***r* = 0.45** ****	*r* = 0.20 * ***r* = 0.44** ****	***r* = 0.36** ** *r* = 0.27 *	*r* = −0.15 *r* = 0.25 **	*r* = 0.01 ***r* = 0.44** ***	***r* = 0.32** * *r* = −0.03	*r* = 0.09 *r* = 0.21	*r* = 0.28 * *r* = 0.08	*r* = −0.17 *r* = −0.02
GLR	*r* = −0.16 *r* = −0.28 **	*r* = 0.22 * ***r* = 0.44** ****	*r* = 0.19 * ***r* = 0.42** ****	***r* = 0.37** ** *r* = 0.27 *	*r* = −0.18 *r* = 0.23 *	*r* = −0.05 ***r* = 0.43** ***	*r* = 0.28 * *r* = −0.03	*r* = 0.10 *r* = 0.20	*r* = 0.28 * *r* = 0.07	*r* = −0.16 *r* = −0.04
PLR	***r* = −0.44** **** *r* = −0.08	*r* = 0.24 ** *r* = 0.25 **	***r* = 0.35** **** *r* = 0.13	***r* = 0.36** ** *r* = 0.13	*r* = 0.10 *r* = 0.07	*r* = 0.13 ***r* = 0.42** ***	*r* = 0.19 *r* = −0.01	*r* = 0.20 *r* = 0.12	*r* = 0.09 *r* = −0.05	*r* = −0.16 *r* = 0.23 *

*Note:* Eighteen women (pink) and 19 men (blue) were subjected to five days of DI. Pearson's correlation coefficient for normally distributed data or Spearman's correlation coefficient for nonnormally distributed data were assessed at the BDC‐4, BDC‐1, DI1, DI3, DI5, and R+1 time points for DI‐associated stress, at the BDC, DI5, and R+1 time points for psychological state, and at the BDC‐1, DI3, and DI5 time points for stress, inflammation, the atherogenic index of plasma, and endothelial markers. The threshold for significance was *p* < 0.05. Statistically significant correlations with *r* values between ±0.30 and ±0.5 were considered as moderate (bold and underlined), and those with *r* values below ±0.30 as weak.

Abbreviations: BDC, baseline data collection; DI, dry immersion; R, recovery.

* *p* < 0.05.

** *p* < 0.01.

*** *p* < 0.001.

**** *p* < 0.0001.

We also assessed psychological state at BDC‐4, DI4, R0, and R+1 using the General Health Questionnaire GHQ‐28. GHQ‐28 scored 1.8 ± 0.4 in men and women at BDC‐4, increased to 8.5 ± 0.9 for women and 6.5 ± 1.2 for men at DI4, remained statistically higher than baseline at R0 (5.5 ± 0.8) for men but not women, and returned to pre‐immersion values (2.1 ± 0.9 for women and 1.6 ± 0.6 for men) at R+1 (Figure 3D). Correlation search revealed statistically significant moderate positive relationships between all ratios (NLR, GLR and PLR) and the GHQ‐28 score in women (Table 1). For men, statistically significant weak positive relationships were observed between NLR, GLR, and GHQ‐28 scores.

### 3.3 Relationships Between NLR, GLR, PLR, Soluble Markers of Endothelial Status, UFC and hsCRP During Dry Immersion

Given that 7 days of DI have been shown to disturb endothelial functions in men [17] and that stress can increase neutrophil count [18] which is associated with inflammation [19], we quantified soluble markers of endothelial status, urinary free cortisol (UFC), high‐sensitivity C‐reactive protein (hsCRP), and searched for correlations between the evolution of NLR, GLR, PLR, and these markers. Furthermore, as higher cardiovascular disease mortality was observed in Apollo lunar astronauts [20] and elevated NLR and PLR are predictive of cardiovascular disease risk [12, 13], as the atherogenic index of plasma (AIP) [14], we studied AIP and searched for correlations between changes in NLR, GLR, and PLR and those in AIP.

**FIGURE 4:**
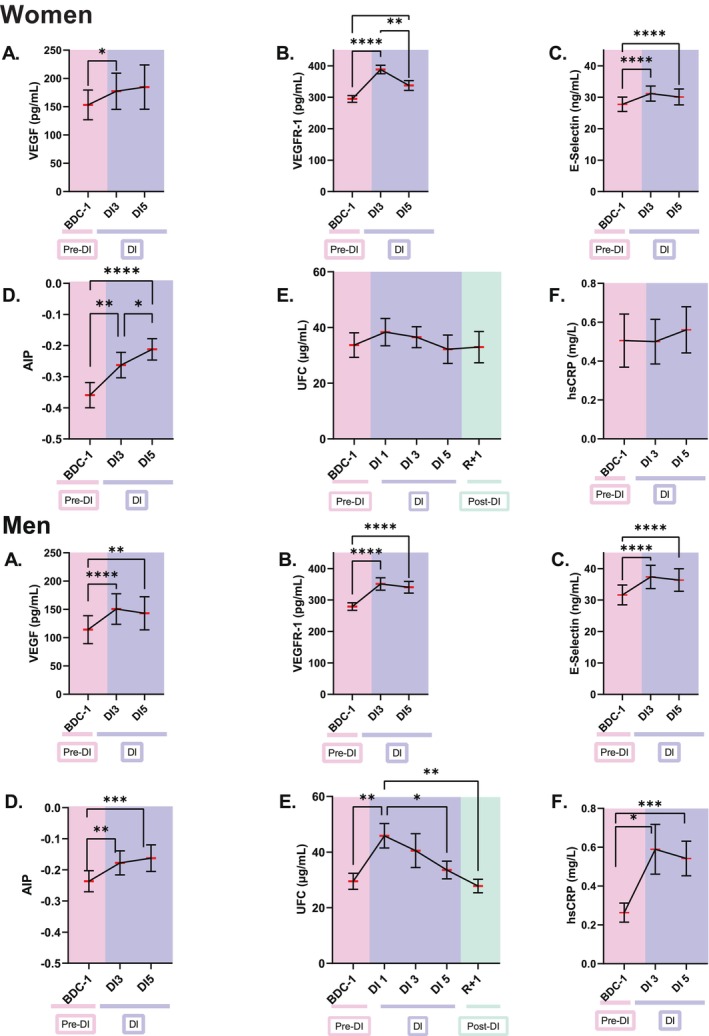
Evolution of VEGF (A), VEGFR‐1 (B), E‐selectin (C), AIP (D), UFC (E) and hsCRP (F) in women (*n* = 18) and men (*n* = 19) exposed to five days of dry immersion. Note that we could not quantify VEGF, VEGFR‐1, E‐selectin, AIP and hsCRP at BDC‐4, DI1 and R+1, and UFC at BDC‐4. The data are shown as mean ± SEM. Statistically significant differences were revealed via repeated‐measures one‐way ANOVA or linear mixed effects model analysis when some data were missing, both of which were followed by a Tukey's *post hoc* test. *p < 0.05; **p < 0.01; ***p < 0.001; ****p < 0.0001. BDC, baseline data collection (pink); DI, dry immersion (blue); R, recovery (green).

Statistically significant increases in vascular endothelial growth factor (VEGF), VEGF‐receptor 1 (VEGF‐R1) and E‐selectin, expressed on endothelial cells after activation, were observed at DI3 in women and men, followed by decreases at DI5 except for VEGF in women (Figure 4). The atherogenic index of plasma (AIP) increased from −0.36 ± 0.04 at BDC‐1 to −0.21 ± 0.03 at DI5 in women, and from −0.24 ± 0.03 at BDC‐1 to −0.16 ± 0.04 at DI5 in men. No significant change in UFC and hsCRP was noted in women while both significantly increased in men, as previously noted by Houreau et al. [21] for hsCRP. Correlation search identified four statistically significant positive relationships in women: a moderate correlation between the evolution of NLR and AIP, a weak correlation between the evolution of GLR and AIP, and weak correlations between the evolution of NLR, GLR, and VEGF‐receptor 1 levels (Table 1). In men, six statistically significant positive correlations were observed: moderate correlations between NLR, GLR, PLR, and hsCRP levels, weak correlations between NLR, GLR, and urinary free cortisol levels, and a weak correlation between PLR and VEGF levels.

Taken together, these data suggest a positive link between NLR and alteration in the cardiovascular system in women, whereas in men, there would be a positive link between NLR, GLR, PLR, and inflammation.

## 4 Discussion

Integrating two kinds of circulating immune cells by measuring ratios such as GLR, NLR, and PLR is a more reliable biomarker than the count of single immune cells and are predictive biomarker for several inflammatory, autoimmune, infectious, and cardiovascular diseases, various types of cancer, and aging [4, 5, 6, 13, 22, 23, 24, 25, 26]. Furthermore, the use of ratios avoids having to take into account microgravity‐induced plasma resorption [21] affecting individual blood cell concentrations, which will facilitate on‐orbit biomedical analyses during future long‐duration space missions.

### 4.1 Real and Simulated Microgravity Increases NLR and GLR

Previous studies revealed significant increases in the GLR ratio after 180 days on‐orbit and in samples collected within 2–3 h post‐landing in 23 astronauts (18 males and 5 females, data for both sexes mixed) and in the NLR ratio after 14 days in‐flight and immediately post‐landing in male rats from the Space Life Sciences (SLS)‐2 mission, followed by readaptation [2]. A similar pattern was observed in human peripheral blood following 20 h of HARV‐RWV modeled microgravity, in female mice after 14 days of hindlimb unloading [2], a model of microgravity that can be considered the rodent equivalent of HDBR [27], and in 20 men one day after the completion of a 2‐month HDBR [3]. Taken together, these data, combined with those presented here, indicate that increases in NLR and GLR are recurrently observed in different species exposed to simulated or real microgravity, regardless of duration, and that sex does not significantly affect these ratios.

In addition, as HARV‐RWV modeled microgravity has been shown to increase apoptosis in human lymphocytes [2], it is possible that such a process occurred during DI because a reduction in lymphocyte concentration was observed. This decrease could also be due to a reduction in B and T lymphopoiesis, as has been observed in humans and mice subjected to real or simulated microgravity [28, 29, 30]. Given the long lifespan of lymphocytes (from a few months to several years) and the limited duration of the DI experiment (5 days), the decrease in lymphocytes is limited in our study. With regard to changes in the concentrations of other cell types, while some studies have shown that myelopoiesis is reduced in real or simulated microgravity (in‐flight collected samples [31] and samples exposed to RPM [32]), another study reported an increase in the proportion of monocyte/macrophage lineage cells in the peripheral blood of mice subjected to simulated microgravity (hindlimb unloading [33]). This last observation is in agreement with the elevated innate immune cell to lymphocyte ratios observed here. A likely explanation for the increase in NLR and GLR is biomechanical stress, as this induces mobilization of innate cells stored in the bone marrow [18], and the peak in neutrophils and granulocytes coincided with the peak in biomechanical stress. Since granulocytes have a short lifespan (from a few hours to a few days), this explanation probably helps to elucidate the larger increases in neutrophils and granulocytes and, consequently, in NLR and GLR ratios. Finally, as observed here, Jacob et al. [3] noted a slight increase in platelets during HDBR, which they attributed to an increase in thrombopoiesis. Since this increase was lower than that of neutrophils and granulocytes, the increases in PLR were less significant than those in NLR and GLR.

### 4.2 DI‐Induced PLR Increase Does Not Presage the Development of Long‐Lasting Immune Diseases, Unlike Two Months of HDBR

We previously noted that PLR increases after 2 months of HDBR [3] to reach a value close to 138 determined in systemic lupus erythematosus patients [25], suggesting the potential development of long‐lasting immune diseases after exposure to this ground‐based model of microgravity. Unfortunately, to our knowledge, this ratio has never been studied during space missions. Even if it is currently unknown whether actual microgravity increases the PLR ratio and there is no clear evidence that spaceflight is associated with a risk of developing autoimmune disease, a few observations suggest that this could be the case, such as (i) the decrease in murine medullary thymic epithelial cells observed after 14 days of hindlimb unloading [34], which are essential for the elimination of autoreactive T lymphocytes and the production of regulatory T lymphocytes [35]; and (ii) the fact that socioenvironmental stressors similar to those encountered during space missions could increase the autoreactivity of the murine TCRβ repertoire [36]. Here, we noted that after 3 days of DI, PLR reached a peak of 137 in women and 129 in men. However, thereafter, this ratio decreased in both men and women, indicating that the potential risk of long‐lasting disease is low. This difference could be explained by the fact that DI‐induced supportlessness and gravitational unloading due to buoyancy are key differences from HDBR and that DI is considered a faster model for simulating the effects of microgravity [16].

### 4.3 Increases in NLR, GLR, and PLR are Associated With Physical and Psychological Stress but Not With Major Inflammation During DI

We previously suggested that an increase in NLR and GLR could be a biomarker of physical stress [3], such as landing or the switch from a prone to an upright position at the end of HDBR. The observation of statistically significant moderate relationships between the evolution of the NLR, GLR, and PLR ratios and those of sleep quality, back pain, and general discomfort used to evaluate DI‐induced biomechanical stress supported the existence of a link between physical stress and changes in these ratios. However, in this study, we observed progressive increases in NLR, GLR, and PLR during, and not at the end or just after DI exposure, indicating adaptation to this unloading model as previously shown for bones during a 5‐day DI [8, 37]. This difference in kinetics could very well be because (i) as indicated above, DI is considered a more rapid model for simulating the effects of microgravity, and (ii) although DI and HDBR are two models that simulate microgravity, these models elicit different biomechanical responses. For example, the cervical region is differently affected by HDBR and DI; furthermore, visceral pain is associated with DI but not HDBR [38]. These biomechanical responses result in more back pain and discomfort during DI [8] than during HDBR [39].

We also noted that the NLR and GLR ratios in male volunteers are statistically greater at BDC‐1 than at BDC‐4 while a non‐significant increase (*p* = 0.1145 for NLR; *p* = 0.1544 for GLR) was observed in women. This could be an effect of the expectation/anxiety to the upcoming DI campaign with men being more apprehensive than women. Sex differences in brain structures and/or cognitive processes may be responsible for this observation. Indeed, male rats are significantly more vulnerable to adult restraint stress, at least in terms of measured emotional behaviors, whereas females are far more resilient [40]. This suggests a psychological contribution to the increases in NLR and GLR prior to DI exposure. This hypothesis is supported by the observation of moderate positive correlations between these two ratios and UFC in men but not in women, by the analyses of UFC levels, which were increased in men but not in women, as well as by psychological state which was longer affected in men than in women and is known to induce a redistribution of immune cells [41].

As indicated above, increases in NLR and GLR are used as predictive biomarkers for several diseases. An elevated NLR (> 3.53) [42] has been implicated in clinical settings to identify heightened inflammation [22]. Neither HDBR [3] nor DI induced such an NLR increase. An NLR value of 3.6 was observed only on the landing day after a 6‐month mission on the ISS [2]. Thus, similar to the transient increase in PLR discussed above, the transient increases in NLR and GLR do not reflect major inflammation. In agreement with this conclusion, cytokine quantification in 12 healthy men of the same age range (31.8 ± 1.4 vs. 28 ± 1 here, mean ± SEM) subjected to 3 days of DI at the same Space Clinic as this study revealed undetectable levels of inflammatory cytokines (IL‐1β, IL‐6, TNFα, IL‐17) [43]. The 2‐month HDBR, which revealed an increase in NLR and GLR at R+1, revealed no evidence of inflammation; it even suggested a potentially lower systemic inflammatory status supported by an increase in serum cortisone [44]. Several independent studies revealed only low‐grade systemic inflammation in astronauts participating in long‐duration ISS missions [45, 46, 47], and serum levels of hsCRP, which reflect acute inflammation, were stable in female participants and within normal ranges in male participants even if significant increases were noted at DI3 and DI5. However, we cannot exclude possible low‐grade systemic inflammation during DI, as previously suggested by Horeau et al. [21], because moderate positive relationships between the NLR, GLR, and PLR ratios and hsCRP levels were observed in male subjects, such as in astronauts who also experience increased levels of C‐reactive protein [48] and who are, thus far, predominantly male.

### 4.4 NLR, GLR and Vascular Markers

Spaceflight most likely damages the endothelium. Indeed, an increase in the plasma levels of von Willebrand factor, which is mainly secreted by the endothelium, was observed after spaceflight in 15 male cosmonauts who had completed orbital flights lasting from 115 to 205 days [48]. Increases in the number of circulating endothelial cells, an index of endothelial damage, were noted after 2 months of HDBR [49] and 7 days of DI were shown to affect endothelial function in men [16]. Given the interplay between endothelial and immune cells, we quantified soluble markers of endothelial status, as well as AIP.

Increases in VEGF and VEGF‐R1 were observed during DI, indicating a likely change in the balance between endothelial cell survival and apoptosis [50]. VEGF conveys signals that promote survival and proliferation in endothelial cells, thereby enhancing their anti‐apoptotic properties. In contrast, VEGF‐R1 sequesters VEGF from signaling receptors and forms non‐signaling complexes with VEGF‐R2, acting as a natural endogenous inhibitor of VEGF. Increases in soluble E‐selectin, suggesting endothelial activation [51], and of the atherogenic index of plasma (AIP), predictive of cardiovascular risk [14], were also observed. Taken together, these data suggest endothelial alteration, in agreement with previous studies showing that dry immersion can quickly lead to endothelial dysfunction [16, 52].

A moderate positive relationship was observed between the evolution of NLR and AIP in women during DI, suggesting that the increases in women's NLR could be due to a demargination of neutrophils linked to a modification of the endothelium. The absence of such a correlation in men could be due to sex‐specific effects of chronic stressors on cardiovascular function [53]. Indeed, previous studies have reported that restraint stress and chronic stressors decrease estradiol levels and their effects [54, 55, 56, 57], thus decreasing the cardiovascular protective effects of estrogens. Furthermore, a sharp decrease in plasma progesterone was reported after 5 days of DI in healthy women [58]. DI duration, which is probably too short in this study, is another possibility, as Navasiolava et al. [16] reported changes in male endothelial functions in response to a 7‐day DI. If future studies confirm that there are changes in endothelial function in men during longer DI and that these changes can be associated with increases in the NLR, this ratio could perhaps be utilized as an easy‐to‐determine biomarker for monitoring the state of the vascular system during space missions.

## 5 Limitations

Our study is limited by the duration of DI exposure (5 days) and by the fact that we could not quantify hsCRP, E‐selectin, AIP, VEGFR‐1, and VEGF at BDC‐4, DI1, and R+1, UFC at BDC‐4, and assess psychological state at DI1 and DI3. Moreover, additional endothelial integrity markers, such as hyaluronan or syndecan, would have been interesting to study. However, research on the effects of 5 days of DI under the same conditions on both sexes and on a significant number of individuals is undeniably a unique feature of our study.

## 6 Conclusion and Perspectives

This study revealed increases in the NLR, GLR, and PLR ratios in both men and women subjected to 5 days of DI, which are associated with physical and psychological stress. Furthermore, our work suggested a positive link between NLR increase and cardiovascular system alteration in women, whereas in men, there would be a positive link between NLR, GLR, PLR, and inflammation. This suggests that, in addition to physical and psychological stress, sex‐specific factors could contribute to increases in NLR, GLR, and PLR ratios during DI. Further studies will be needed to precise the mechanisms underlying increases in NLR, GLR, and PLR ratios, define space‐appropriate NLR, GLR, and PLR thresholds, and confirm the predictive utility of these ratios to monitor health during spaceflight [7, 28, 59]. Given recent advances in space biotechnology for monitoring peripheral blood cells [60, 61], we believe that these ratios could, in the near future, be monitored onboard spacecraft participating in future deep space missions.

## Author Contributions

P.J., A.R., N.N., and S.G. collected and analyzed the data. N.N. and M.‐A.C. were leaders in the investigator team across the VIVALDI studies. M.‐P.B. and R.B.D.V. coordinated the study and experiments by the medical and paramedical teams at the MEDES clinic. I.A. and A.V.O. designed the VIVALDI studies. G.G.‐K. represented the promoter of the study. J.‐P.F. conceived the data analyses. P.J. and J.‐P.F. were major contributors to the writing of the manuscript. All authors reviewed, edited, and approved the manuscript.

## Conflicts of Interest

The authors declare no conflicts of interest.

## Data Availability

The data that support the findings of this study are available in the Materials and Methods, and Results sections of this article.
